# Amateur Printing

**Published:** 1875-08

**Authors:** 


### AMATEUR PRINTING.

With one of Joseph Watson's Young America presses, sold at his warehouses, 73 Cornhill, Boston, and 53 Murray street, New York, it is not difficult for any boy, or girl, or man, or woman to do good printing. Just as clean and perfect impressions, and quite as elegant work can be done on these presses as on any others ever made. There are "lightning" presses which can do faster work, but we have never seen finer work done than has been produced by intelligent boys on a Young America press. An equally important fact is that these little presses are durable; they do not wear out. Their builder is a practical man who knows exactly where the strain comes in a press, and right there he puts the strength.

Now, we want to tell parents that there is no educational gift which we can deem as valuable for a child of either sex, as one of these printing-presses. By its use a very fair education can be gained. The little printer acquires a knowledge of spelling, of composition, and of proof-reading, while at the same time learning a trade by which a living can be made anywhere. When we consider that for the sum of seven dollars and twenty-five cents a complete printing office can be bought and delivered free by mail, wherever the mail goes. The press sent for this sum is called the Centenial Press, the form measuring two and a quarter by three and a quarter inches. It is a strong, elegant little affair, and costs here only $2, with ink-rollers. It is a perfect little device for cards or small labels or circulars. Mr. Watson is its inventor, as he is the inventor of the leading improvements in all his Young America presses, which are, in our judgment, the best small presses made. He sends a very interesting pamphlet, with cuts and specimens of type, for ten cents.

